# Sarcopenia and dosimetric parameters in relation to treatment-related leukopenia and survival in anal cancer

**DOI:** 10.1186/s13014-021-01876-5

**Published:** 2021-08-16

**Authors:** Martin P. Nilsson, Anders Johnsson, Jonas Scherman

**Affiliations:** 1 Division of Oncology and Pathology, Department of Clinical Sciences, Lund University, Lund, Sweden; 2 Department of Hematology, Oncology and Radiation Physics, Skåne University Hospital, Lasarettsgatan 23, 221 85 Lund, Sweden; 3 Radiation Physics, Department of Hematology, Oncology and Radiation Physics, Skåne University Hospital, Lund, Sweden

**Keywords:** Anal cancer, Anal carcinoma, Leukopenia, White blood cell, Dosimetric parameters, Sarcopenia

## Abstract

**Background:**

Treatment-related white blood cell (WBC) toxicity has been associated with an inferior prognosis in different malignancies, including anal cancer. The aim of the present study was to investigate predictors of WBC grade ≥ 3 (G3+) toxicity during chemoradiotherapy (CRT) of anal cancer.

**Methods:**

Consecutive patients with locally advanced (T2 ≥ 4 cm—T4 or N+) anal cancer scheduled for two cycles of concomitant 5-fluorouracil and mitomycin C chemotherapy were selected from an institutional database (*n* = 106). All received intensity modulated radiotherapy (IMRT; mean dose primary tumor 59.5 Gy; mean dose elective lymph nodes 45.1 Gy). Clinical data were extracted from medical records. The highest-grade WBC toxicity was recorded according to CTCAE version 5.0. Pelvic bone marrow (PBM) was retrospectively contoured and dose-volume histograms were generated. The planning CT was used to measure sarcopenia. Dosimetric, anthropometric, and clinical variables were tested for associations with WBC G3+ toxicity using the Mann–Whitney test and logistic regression. Cox proportional hazard regression was used to assess predictors for overall survival (OS) and anal cancer specific survival (ACSS).

**Results:**

WBC G3+ was seen in 50.9% of the patients, and 38.7% were sarcopenic. None of the dosimetric parameters showed an association with WBC G3+ toxicity. The most significant predictor of WBC G3+ toxicity was sarcopenia (adjusted OR 4.0; *P* = 0.002). Sarcopenia was also associated with an inferior OS (adjusted HR 3.9; *P* = 0.01), but not ACSS (*P* = 0.07). Sensitivity analysis did not suggest that the inferior prognosis for sarcopenic patients was a consequence of reduced doses of chemotherapy or a prolonged radiation treatment time. Patients who experienced WBC G3+ toxicity had an inferior OS and ACSS, even after adjustment for sarcopenia.

**Conclusions:**

Sarcopenia was associated with increased risks of both WBC G3+ toxicity and death following CRT for locally advanced anal cancer. In this study, radiation dose to PBM was not associated with WBC G3+ toxicity. However, PBM was not used as an organ at risk for radiotherapy planning purposes and doses to PBM were high, which may have obscured any dose–response relationships.

**Supplementary Information:**

The online version contains supplementary material available at 10.1186/s13014-021-01876-5.

## Introduction

Leukopenia—defined as low white blood cell (WBC) levels—is a common side effect to chemoradiotherapy (CRT) of anal cancer [1, 2]. Patients with severe leukopenia are at risk of life-threatening complications such as febrile neutropenia and neutropenic enterocolitis. Also, WBC toxicity may lead to dose reductions, delays of chemotherapy, and prolonged overall treatment time, potentially diminishing the chance of cure [3]. Moreover, functional WBCs are important for the antitumor effect. Radiation-induced lymphopenia and leukopenia has been shown to be associated with inferior outcomes in several types of malignancies, including anal cancer [4–7]. One possible way of reducing WBC toxicity is to reduce the radiation dose to the pelvic bone marrow (PBM). However, previous studies on the associations between PBM dosimetric parameters and WBC toxicity have shown conflicting results [8–16]. Another factor of potential importance for WBC toxicity is sarcopenia (loss of skeletal muscle mass) [17]. In a recent study by Martin et al., sarcopenic patients had an increased risk of hematologic toxicity during anal cancer CRT [18]. To the best of our knowledge, no previous study has investigated the effect of both PBM dosimetric parameters and sarcopenia on hematologic toxicity.

The aim of the present study was to analyze potential predictors of WBC grade ≥ 3 (G3+) toxicity during CRT of locally advanced anal cancer. In addition to clinical variables, the importance of dosimetric parameters and sarcopenia was also assessed.

## Material and methods

### Study population and treatment

**Fig. 1:**
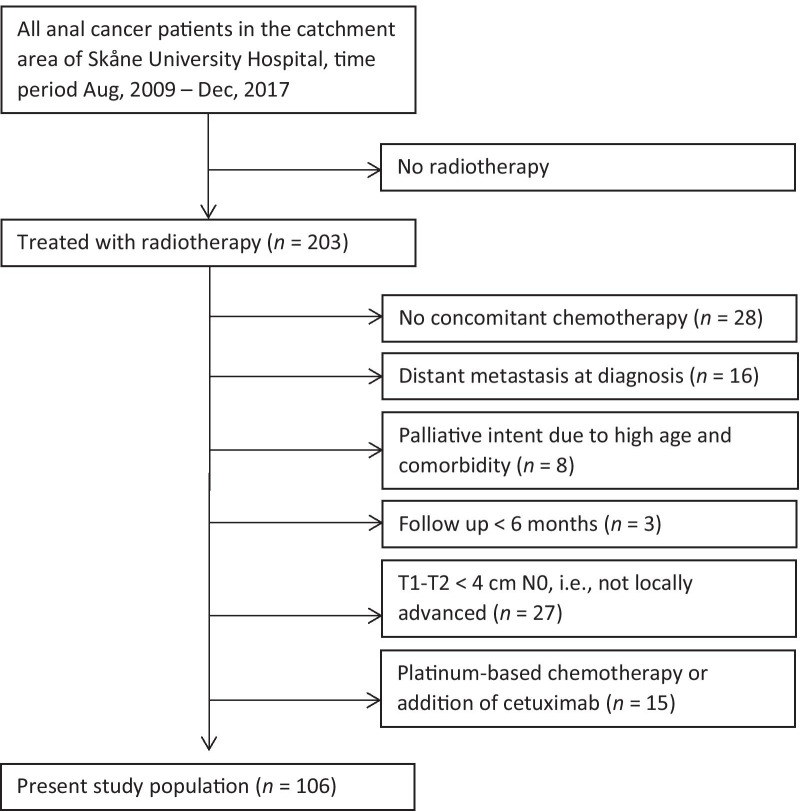
Flowchart of the study population

The study population and data collection has been described in detail previously [7, 19]. Briefly, all patients with squamous cell carcinoma of the anus (anal cancer) treated with radiotherapy at the Skåne University Hospital, Lund, Sweden, during the years 2009–2017 were selected from an institutional database. For the present study, only patients with locally advanced nonmetastatic disease (T2 ≥ 4 cm—T4 or N+) scheduled for 5-fluorouracil and mitomycin C concomitant chemotherapy were included (Fig. 1). The reason for excluding patients with T1–T2 < 4 cm N0 disease was that they were treated with *both* lower doses of radiotherapy *and* less chemotherapy, confounding dosimetric analyses. Treatment was according to Swedish national guidelines, and all received intensity modulated radiotherapy (IMRT). Before 2017, the prescribed dose to the primary tumor and lymph node metastasis was 60 Gy/30 fractions (F), and the prescribed dose to the elective clinical target volume (CTV) was 46 Gy/23F. For patients treated in 2017 (*n* = 12), the following prescribed doses were used: primary tumor and lymph node metastasis ≥ 4 cm 57.5 Gy/27F; lymph node metastasis < 4 cm 50.5 Gy/27F; elective CTV 41.6 Gy/27F. Concomitant chemotherapy consisted of two cycles of mitomycin (10 mg/m^2^ on day 1 and 29) and 5-fluorouracil (1000 mg/m^2^ on days 1–4 and 29–33). Radiotherapy was given without planned treatment breaks, and granulocyte-colony stimulating factor was not routinely used. It was recommended that the contouring of the elective CTV should be in line with the RTOG guidelines [20]. PBM was not used as an organ at risk or an optimization structure during the radiotherapy planning process. The study was approved by the Regional Ethical Review Board in Lund (Dnr 2013/742).

### Data collection

**Fig. 2:**
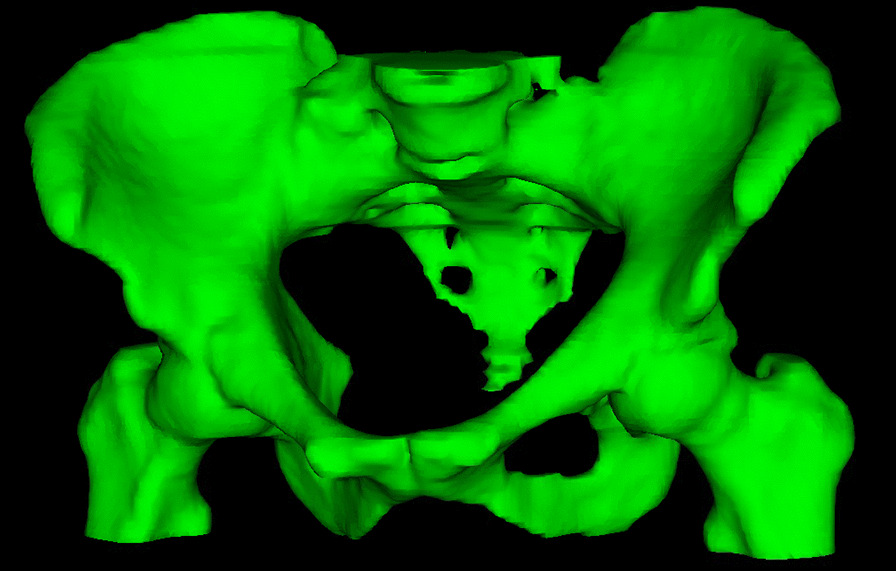
Pelvic bone marrow

Data were extracted from medical records. WBC count was measured routinely before commencement of CRT, weekly during treatment, and post-treatment when clinically indicated. Acute (within 90 days after the end of radiotherapy) toxicity was graded according to CTCAE version 5.0. The highest-grade WBC toxicity was recorded. For the present study, dosimetric and anthropometric data, including sarcopenia, were added to the clinical data from our previous publications [7, 19]. PBM was retrospectively contoured in accordance with Mell et al. [13]. The external contour of bones was auto-segmented in Eclipse v 15.6 (Varian Medical Systems, Palo Alto, CA, USA) using a range of 100 to maximum Hounsfield units and 3D processing mode with asymmetric smoothing of 1. A volume of interest was used to limit the auto-segment to include all bones from the superior border of the L5 vertebral body to the inferior border of the ischial tuberosities (Fig. 2). The cranial parts of the iliac crests were manually segmented if they were not included in the auto-segmentation. Post processing using smoothing level 2 and fill all cavities was used after possible manual adjustment, which could be for example due to the auto-segmentation failed to include all the external contour of the pelvic bone or due to contrast agents or atherosclerosis.

Dose-volume histograms were generated for PBM, and the following parameters were recorded: mean dose (Dmean), the percentage of PBM volume receiving at least 10–50 Gy in 10 Gy increments (V10–50 Gy), and the absolute volume of PBM receiving less than 10–50 Gy in 10 Gy increments (V < 10–50 Gy). Body surface area (BSA) was calculated using the Du Bois method [21]. Dose-volume data was retrieved using Eclipse scripting API (ESAPI v 15.6, Varian Medical Systems, Palo Alto, CA, USA).

**Fig. 3:**
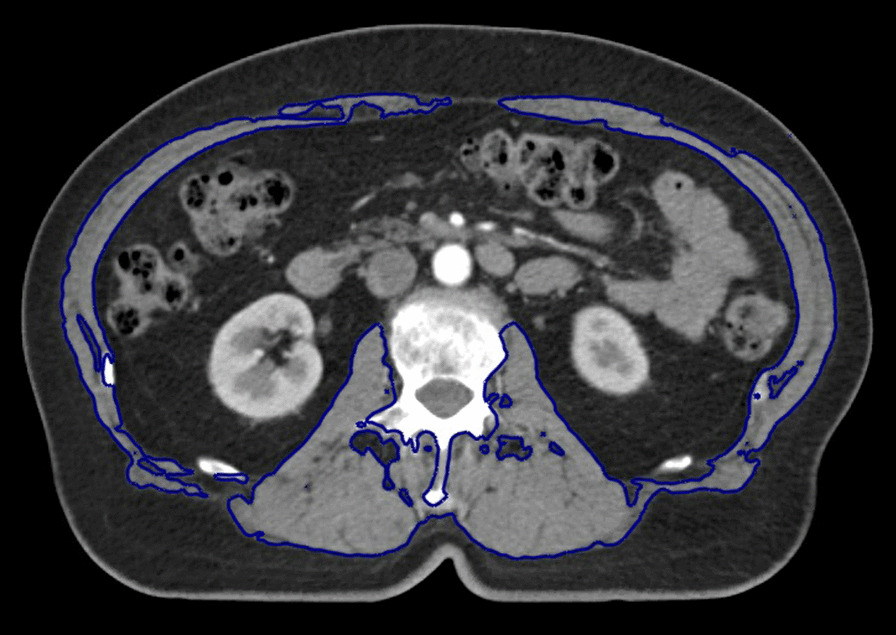
Skeletal muscle at the level of L3

The planning CT was used to measure sarcopenia. A single axial CT slice at the level of the L3 transverse process was selected. Skeletal muscle was first auto-segmented using a range of − 29 to 150 Hounsfield units, and then manually adjusted to exclude all non-muscle tissues (Fig. 3). The area of the segmented skeletal muscles was retrieved by multiplying the number of pixels included in the segmentation with the pixel size and dividing this value with the CT slice thickness. Skeletal muscle index (SMI) was calculated as the area of skeletal muscle divided by patient height squared. SMI thresholds of sarcopenia were 38.5 cm^2^/m^2^ for women and 52.4 cm^2^/m^2^ for men. For some patients, the planning CT scan did not extend to the L3 level. Instead, a single CT slice at the most inferior aspect of L4 was used, in accordance with Martin et al. [18]. Thresholds of sarcopenia for these patients were 34.2 cm^2^/m^2^ for women and 41.3 cm^2^/m^2^ for men. Both the L3 and the L4 cut-offs are well established from previous studies [18, 22].

### Statistical analysis

For categorical variables, proportions were compared by the Chi square test or Fisher’s exact test, as appropriate. For continuous dosimetric and anthropometric variables, the values for patients with G0-2 WBC toxicity were compared with the values for patients with G3-4 WBC toxicity using the Mann Whitney test. Variables with *P* < 0.05 from these analyses were used together with clinical variables in logistic regressions of factors correlated with an increased risk of WBC G3+ toxicity. Pearson correlation coefficient was used to analyze bivariate correlations between two continuous variables. Multicollinearity was analyzed using variance inflation factors (VIF).

Overall survival (OS) and anal cancer specific survival (ACSS) were analyzed. Follow-up and time to endpoint was defined from the date of diagnosis. Survival was estimated by the Kaplan–Meier method and compared using the log rank test. Cox proportional hazards model was used to assess predictors for survival endpoints.

Predicting variables with a significance of *P* < 0.10 in univariate analysis for a certain endpoint were entered into a multivariate logistic regression or Cox model for that endpoint. Age at diagnosis, pretreatment leukocyte count, pretreatment hemoglobin, and tumor size were treated as continuous variables. For time to treatment initiation, and total radiation treatment time, previously defined cut-offs were used [7]. All significance tests were 2-sided, and *P* values < 0.05 were considered statistically significant. Statistical analysis was conducted using SPSS version 25 (SPSS Inc., Chicago, Illinois, USA).

## Results

One-hundred and six patients with locally advanced anal cancer, treated with curative intent IMRT (mean dose primary tumor 59.5 Gy; mean dose elective lymph nodes 45.1 Gy) and concomitant chemotherapy, were included in the study. Patient, tumor, and treatment characteristics are listed in Table 1. Median age at diagnosis was 63.8 years, and 79.2% were female. SMI was measured at L3 (*n* = 94) or at L4 (*n* = 12). In total, 41 of 106 (38.7%) were sarcopenic. WBC G3+ toxicity (50.9%) and febrile neutropenia (23.7%) were relatively frequent side effects. Five-year OS was 82.5% and five-year ACSS was 88.9%.

**Table 1:** Patient, tumor, and treatment characteristics

	*n* (%)
Age at diagnosis (years), Median; range	63.8; 44.1–82.7
Female gender	84 (79.2)
Active smoking	32 (30.2)
Charlson comorbidity index ≥ 1	44 (41.5)
Sarcopenia	41 (38.7)
Immunosuppressive disorders^a^	12 (11.3)
HIV	1 (0.9)
Pretreatment hemoglobin (g/L), Median; range	129; 77–163
Pretreatment leukocyte count (10^9^/L), Median; range	7.6; 3.8–33.7
T stage^b^	
1	3 (2.8)
2	46 (43.4)
3	27 (25.5)
4	30 (28.3)
Primary tumor size (cm), Median; range	5.0; 1.0–15.0
Lymph node metastasis^b^	67 (63.2)
Radiation technique	
IMRT	9 (8.5)
Tomotherapy	20 (18.9)
VMAT	77 (72.6)
Radiation dose (Gy)	
Primary tumor, Mean; SD	59.5; 1.2
Lymph node metastasis, Mean; SD	57.9; 4.4
Elective, Mean; SD	45.1; 4.0
Radiation treatment time (days), Mean; SD	43.1; 4.6
Included in elective CTV^c^	
Inguinal	104 (98.1)
Internal iliac	106 (100.0)
External iliac	104 (98.1)
Presacral	106 (100.0)
Mesorectal	106 (100.0)
Ischiorectal fossa	101 (95.3)
Low cranial border^d^ of elective CTV	8 (7.5)
Chemotherapy: all patients scheduled for FUMI × 2	
Omission of second cycle	14 (13.2)
Dose reduction of second cycle	15 (14.2)
Omission *or* dose reduction of second cycle	29 (27.4)
Second cycle platinum-based (cardiac toxicity)	4 (3.8)
White blood cell toxicity (CTCAE version 5.0)	
G0	15 (14.2)
G1	7 (6.6)
G2	30 (28.3)
G3	36 (34.0)
G4	18 (17.0)
Febrile neutropenia	22 (20.8)

*cc* cubic centimeter, *CTV* clinical target volume, *FUMI* 5-fluorouracil + Mitomycin C, *G* grade, *Gy* gray, *IMRT* intensity modulated radiation therapy, *SD* standard deviation, *VMAT* volumetric modulated arc therapy

^a^Connective tissue disorder (*n* = 6), inflammatory bowel disease (*n* = 4), chronic leukemia (*n* = 1), heart transplant (*n* = 1)

^b^TNM8

^c^ > 80% of region covered in elective CTV to count as ‘included’

^d^ ≥ 3 cm below sacral promontory (8 patients: median 36 mm; range 30–42 mm)

### Dosimetric and anthropometric variables

None of the dosimetric variables showed an association with WBC G3+ toxicity (Table 2). The only anthropometric variable that was significantly associated with WBC G3+ toxicity was PBM volume (*P* = 0.041). Consequently, only PBM volume was used for further analyses, together with clinical variables and sarcopenia (Table 3). PBM volume was correlated with many other variables in Table 2, e.g., with dosimetric parameters PBM V < 20 Gy (r = 0.51; *P* < 0.001), PBM V < 30 Gy (r = 0.67; *P* < 0.001), PBM V < 40 Gy (r = 0.81; *P* < 0.001), and with height (r = 0.86; *P* < 0.001).

**Table 2:** Associations between dosimetric/anthropometric variables and white blood cell grade ≥ 3 toxicity (median values reported)

	All patients (*n* = 106)	WBC G3+ toxicity		
		No (*n* = 52)	Yes (*n* = 54)	*P*
*Dosimetric variables*				
PBM				
Dmean (Gy)	32.0	32.1	31.9	0.390
V10Gy (%)	87.3	86.1	88.1	0.574
V20Gy (%)	79.5	78.7	80.0	0.733
V30Gy (%)	61.0	62.1	60.4	0.742
V40Gy (%)	35.5	36.6	34.9	0.242
V50Gy (%)	7.8	8.4	6.2	0.054
V < 10 Gy (cc)	174	185	149	0.373
V < 20 Gy (cc)	294	306	277	0.397
V < 30 Gy (cc)	547	557	536	0.552
V < 40 Gy (cc)	879	912	843	0.356
V < 50 Gy (cc)	1263	1291	1220	0.255
PTV (cc)	2614	2609	2624	0.604
PTV outside PBM (cc)	2435	2423	2446	0.570
*Anthropometric variables*				
PBM volume (cc)	1363	1406	1330	**0.041**
Height (cm)	167	167	166	0.069
Weight (kg)	69.1	72.5	68.0	0.229
BMI (kg/m^2^)	25.2	24.9	25.7	0.845
BSA (m^2^)	1.78	1.81	1.77	0.133

Bold indicates *P*-value < 0.05

*BMI* body mass index, *BSA* body surface area, *WBC* white blood cell, *PBM* pelvic zone marrow, *PTV* planning target volume

**Table 3:** Logistic regressions of predictors for white blood cell grade ≥ 3 toxicity

Variable	WBC G3+ toxicity			
	Univariate		Multivariate^a^	
	OR (95% CI)	*P*	OR (95% CI)	*P*
Female gender	2.7 (1.0–7.4)	0.049	3.2 (0.6–16.4)	0.17
Active smoking	0.8 (0.3–1.8)	0.58	.	.
Charlson comorbidity index ≥ 1	1.1 (0.5–2.4)	0.82	.	.
Immunosuppressive disorders	2.1 (0.6–7.4)	0.26		.
Sarcopenia	3.2 (1.4–7.4)	0.005	4.0 (1.6–9.8)	0.002
Age at diagnosis	1.02 (0.98–1.07)	0.40	.	.
Pretreatment leukocyte count	0.92 (0.82–1.02)	0.103	.	.
Pretreatment hemoglobin	0.99 (0.97–1.02)	0.52	.	.
PBM volume^b^	0.86 (0.72–1.02)	0.08	0.96 (0.73–1.27)	0.79

*CI* confidence interval, *OR* odds ratio, *PBM* pelvic bone marrow, *WBC* white blood cell

^a^Pseudo R square for the multivariate model: Nagelkerke (0.17), Cox & Snell (0.13)

^b^OR per 100 cc increase

### Predictors of WBC G3+ toxicity

In univariate analysis, females (OR 2.7; *P* = 0.049) and sarcopenic patients (OR 3.2; *P* = 0.005) had an increased risk of WBC G3+ toxicity (Table 3). In a multivariable model, that also included PBM volume, only sarcopenia retained its statistical significance (OR 4.0; *P* = 0.002). However, PMB volume was smaller in females than in males (median 1315 *vs* 1765 cc; *P* < 0.001) and the nonsignificant adjusted odds ratios for these two variables should be interpreted in the light of some—although not major—problems with multicollinearity (VIF = 2.39).

### Sarcopenia and survival

In Additional file 1: Table S1, characteristics of sarcopenic and non-sarcopenic patients are compared. Sarcopenic patients were older (median 68.2 vs. 61.9; *P* = 0.01), had a lower BMI (median 22.7 vs. 26.7; *P* = 0.001), and more often had the second cycle of chemotherapy cancelled or dose reduced (41 vs. 18%; *P* = 0.01). No significant differences were found regarding gender, BSA, tumor size, radiation treatment time, or any dosimetric parameters. Sarcopenia was not significantly associated with acute or late gastrointestinal toxicity (*P* > 0.10).

**Fig. 4:**
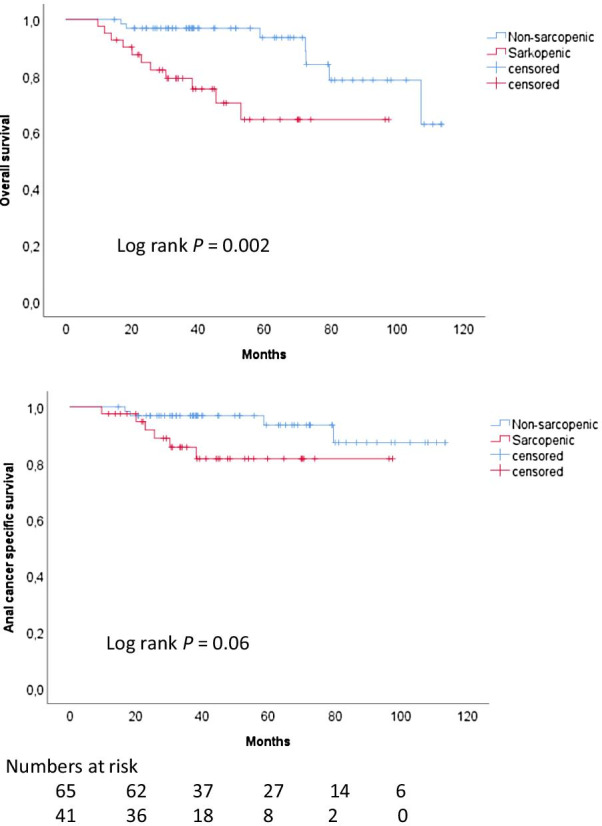
Kaplan–Meier curves of overall survival and anal cancer specific survival for non-sarcopenic versus sarcopenic patients

Sarcopenia was a significant predictor for OS in univariate analysis (HR 4.5; *P* = 0.004) (Table 4). The first multivariate model (Model 1) included all variables with a significance of *P* < 0.10 in univariate analysis. Accordingly, WBC G3+ toxicity was included, and it was associated with an inferior survival (HR 4.4; *P* = 0.02). As sarcopenia was significantly correlated with WBC G3+ toxicity (Table 3), the variable WBC G3+ toxicity was excluded from Model 2 to avoid overadjustment bias (i.e., control for an intermediate variable). In Model 2, sarcopenia was significantly associated with an inferior OS (HR 3.9, *P* = 0.01). No significant association (*P* = 0.07) was seen between sarcopenia and ACSS. In Fig. 4, Kaplan–Meier curves for OS and ACSS are shown comparing sarcopenic and non-sarcopenic patients.

**Table 4:** Univariate and multivariate Cox analyses

Variable	Overall survival						Anal cancer specific survival					
	Univariate		Multivariate				Univariate		Multivariate			
			Model 1		Model 2^a^				Model 1		Model 2^a^	
	HR	*P*	HR	*P*	HR	*P*	HR	*P*	HR	*P*	HR	*P*
Male gender	3.0	0.03	5.4	**0.002**	3.4	**0.02**	3.1	0.08	5.4	**0.02**	2.8	0.12
Active smoking	0.7	0.58	.	.	.	.	0.2	0.16	.	.	.	.
Charlson comorbidity index ≥ 1	1.5	0.42	.	.	.	.	1.2	0.82	.	.	.	.
Immunosuppressive disorders	2.2	0.16	.	.	.	.	3.0	0.11	.	.	.	.
Sarcopenia	4.5	0.004	2.7	0.08	3.9	**0.01**	3.3	0.07	1.8	0.42	3.0	0.10
Age at diagnosis	0.99	0.78	.	.	.	.	0.97	0.36	.	.	.	.
Tumor size (cm)	1.1	0.53	.	.	.	.	1.1	0.42	.	.	.	.
Lymph node metastasis	0.7	0.38	.	.	.	.	1.0	0.97	.	.	.	.
T4	0.9	0.79	.	.	.	.	0.6	0.55	.	.	.	.
White blood cell grade ≥ 3 toxicity	3.3	0.02	4.4	**0.02**	.	.	4.6	0.054	6.7	**0.03**	.	.
Time to treatment initiation ≥ 62 days	1.6	0.37	.	.	.	.	1.5	0.59	.	.	.	.
RTT ≥ 5 days longer than optimal	2.6	0.046	3.2	**0.02**	3.4	**0.02**	2.8	0.12	.	.	.	.
Omission/dose reduction of second cycle of chemotherapy	1.6	0.37	.	.	.	.	1.3	0.68	.	.	.	.

Bold indicates mulvariate *P*-values < 0.05

*HR* hazard ratio, *RTT* radiation treatment time

^a^WBC G3+ toxicity excluded in Model 2

None of the dosimetric parameters listed in Table 2 were correlated with OS or ACSS (all *P* > 0.10, data not shown).

### Sensitivity analysis

Restricting the Cox analysis to patients without dose reduction of the second cycle of chemotherapy (*n* = 77), sarcopenic patients still had an inferior OS (HR 4.2, *P* = 0.02; data not shown). As detailed above, sarcopenia was not significantly associated with radiation treatment time (Additional file 1: Table S1) and retained an association with OS even after adjustment for radiation treatment time (Table 4, Model 2). Taken together, these findings did not suggest that the inferior survival seen in sarcopenic patients was driven by lower doses of chemotherapy or suboptimal delivery of radiotherapy.

## Discussion

We investigated dosimetric, anthropometric, and clinical predictors for WBC G3+ toxicity following CRT in a relatively large cohort of locally advanced anal cancer patients. The most important predictor of WBC G3+ toxicity was shown to be sarcopenia, which was also associated with an inferior OS. Sensitivity analysis did not suggest that the inferior survival for sarcopenic patients was a consequence of reduced doses of chemotherapy or a prolonged radiation treatment time.

Two previous studies have investigated the role of sarcopenia in anal cancer. Martin et al. found that sarcopenic patients had a higher rate of WBC G3+ toxicity but similar survival as non-sarcopenic patients [18]. In contrast, Bingmer et al. reported that sarcopenia was associated with an inferior OS, which is well in line with findings in other malignancies than anal cancer [22, 23]. Interestingly, the results of both our study and the study by Martin et al. suggest that sarcopenia is selectively associated with hematologic toxicity, not gastrointestinal toxicity. The reason for this selectivity in toxicity profile remains unclear, but possible explanations include a reduced total reserve of functional bone marrow in patients with sarcopenia, or common pathophysiological mechanisms causing both deficient hematopoiesis and sarcopenia, e.g., chronic inflammation [18, 24].

In a previous study, we found that WBC G3+ toxicity was associated with an increased risk of recurrence and inferior survival in 170 anal cancer patients [7]. In the present study, which consists of a subset of patients from our previous investigation, adjustment for sarcopenia did not reduce the impact of WBC G3+ toxicity on OS and ACSS.

To the best of our knowledge, our study is the largest to date to analyze dosimetric predictors of hematologic toxicity in anal cancer. In contrast to some previous publications, radiation dose to PBM was not associated with WBC G3+ toxicity. Of note, for the patients included in our study, PBM was not used as an organ at risk during the radiotherapy planning process. Consequently, doses to PBM were high, which may have obscured any dose–response relationships. Indeed, evidence for a myeloprotective effect of bone marrow sparing IMRT (BMS-IMRT) is emerging. In a prospective non-randomized phase II anal cancer study, Arcadipane et al. found that PET-based active BMS-IMRT reduced hematologic toxicity compared to historical data. Overall, 11 out of 39 patients (28%) treated with PET-based active BMS-IMRT experienced G3+ hematologic toxicity and 8 (20%) had WBC G3+ toxicity [25]. The patients in that study, as well the patients in some of the previous retrospective publications, received lower radiation doses to PBM than the patients in our study [8–10]. Other noteworthy differences between our study cohort and previous publications include a high percentage of females (79.2%), a high median age at diagnosis (63.8 years), and exclusion of patients with T1-2N0 tumors and patients not scheduled for two cycles of 5-fluorouracil and mitomycin C concomitant chemotherapy.

A debate is ongoing regarding the optimal dose level to predict hematologic toxicity. Some studies reported high doses (40–50 Gy) [10, 11, 26], whereas other reported lower doses (10–20 Gy) as optimal predictors [8, 13–16]. All studies have been hampered by small sample sizes and high correlations between the dosimetric parameters, making proper statistical analysis hard to carry out and difficult to interpret. In our opinion, the fact that doses in the range of 12–18 Gy are effective for total marrow myeloablation in the treatment of leukemias is favoring the opinion that lower doses are important predictors for hematologic toxicity [27]. In our study, only 20.5% (median value) of PBM received < 20 Gy, making any dose–response relationships between PBM dosimetric parameters and hematologic toxicity hard to discern. Under such circumstances, the remaining bone marrow reserve outside of the pelvis is probably more important than dosimetric distributions within the pelvic bones.

There are limitations to our study. First, we only investigated the role of PBM, not pelvic sub-regions or PET-defined active bone marrow. However, previous studies have not consistently shown that active bone marrow or pelvic-sub-regions are superior to pelvic bones and we therefore decided to focus solely on PBM [16, 28, 29]. Second, the original intention was to measure sarcopenia at the L3 level for all patients. As it turned out that the planning CT did not extend to this level for a minority of the patients, we decided to use the L4 level for these patients, instead of excluding them. In the previous anal cancer studies on sarcopenia, Bingmer et al. used the L3 level, and Martin et al. used the L4 level, without any difference in the prevalence of sarcopenia between the studies (25.0% and 25.4%, respectively) [18, 22]. Third, the association between sarcopenia and survival should be interpreted cautiously in the light of the retrospective nature of our study and the lack of data on some covariates of potential importance. Notably, data on performance status were missing. An association between sarcopenia and performance status has been reported [18], and adjustment for performance status might have influenced on our results.

## Conclusion

Sarcopenia was significantly associated with increased risks of both WBC G3+ toxicity and death following CRT for locally advanced anal cancer. In this study, radiation dose to PBM was not associated with WBC G3+ toxicity. However, PBM was not used as an organ at risk for treatment planning purposes and doses to PBM were high, which may have obscured any dose–response relationships.

## Supplementary Information

**Additional file 1: Table S1**. Sarcopenic versus non-sarcopenic patients.

## Data Availability

The present data is summarized in this paper. The complete dataset can be retrieved from the corresponding author on reasonable request.

## Abbreviations

ACSS: anal cancer specific survival
BSA: body surface area
BMS: bone marrow sparing
CRT: chemoradiotherapy
CTV: clinical target volume
F: fractions
G: grade
IMRT: intensity modulated radiotherapy
OS: overall survival
PBM: pelvic bone marrow
SMI: skeletal muscle index
VIF: variance inflation factors
WBC: white blood cell
