# Supplementary material for: Sarcopenia and dosimetric parameters in relation to treatment-related leukopenia and survival in anal cancer
Source: Radiat Oncol. 2021 Aug 16;16:152. doi: 10.1186/s13014-021-01876-5 (PMC8365937; doi:10.1186/s13014-021-01876-5)
Supplement: Supplementary file 1 — Additional file 1: Table S1. Sarcopenic versus non-sarcopenic patients. [file 13014_2021_1876_MOESM1_ESM.pdf]

Supplementary Table S1. Sarcopenic *vs* non-sarcopenic patients

	Sarcopenia		
	No, *n* (%)	Yes, *n* (%)	P
**Categorical variables**			
Gender			0.22
Male	11 (17%)	11 (27%)	
Female	54 (83%)	30 (73%)	
Active smoking			0.14
No	42 (65%)	32 (78%)	
Yes	23 (35%)	9 (22%)	
Charlson comorbidity index			0.11
0.	42 (65%)	20 (49%)	
≥1	23 (35%)	21 (51%)	
Primary tumor stage			**0.04**
T1-3	42 (65%)	34 (83%)	
T4	23 (35%)	7 (17%)	
Nodal stage			0.43
N0	22 (34%)	17 (42%)	
N+	43 (66%)	24 (58%)	
Immunosupression			0.82
No	58 (89%)	36 (88%)	
Yes	7 (11%)	5 (12%)	
Omission or dose reduction of second cycle			**0.01**
No	53 (82%)	24 (59%)	
Yes	12 (18%)	17 (41%)	
Time to treatment initiation			0.36
<62 days	49 (75%)	34 (83%)	
≥62 days	16 (25%)	7 (17%)	
RTT ≥5 days longer than optimal			0.12
No	53 (82%)	28 (68%)	
Yes	12 (18%)	13 (32%)	
Acute WBC toxicity			**0.01**
G0-2	39 (60%)	13 (32%)	
G3	26 (40%)	28 (68%)	
Acute gastrointestinal toxicity			0.13
G0-2	40 (62%)	19 (46%)	
G3	25 (38%)	22 (54%)	
Late gastrointestinal toxicity			0.67
G0-2	35 (61%)	17 (57%)	
G3	22 (39%)	13 (43%)	
**Continous variables (median values reported)**			
Age at diagnosis (years)	61.9	68.2	**0.013**
RTT (days)	43.0	43.0	0.065
Pretreatment hemoglobin (g/L)	128	131	0.695
Pretreatment leukocyte count (10^9/L)	7.3	7.9	0.509
Tumor size (cm)	5.0	5.5	0.131
Height (cm)	166	167	0.272
Weight (kg)	74.0	65.0	**0.032**
BMI (kg/m2)	26.7	22.7	**0.001**
BSA (m2)	1.82	1.71	0.167

PBM volume (cc)	1365	1357	0.778
PTV (cc)	2615	2598	0.899
PTV outside PBM (cc)	2445	2420	0.915
**Dosimetric variables (median values reported)**			
PBM Dmean (Gy)	32.0	32.0	0.894
PBM V10Gy (%)	87.0	88.0	0.864
PBM V20Gy (%)	79.2	80.0	0.941
PBM V30Gy (%)	61.2	60.2	0.788
PBM V40Gy (%)	36.1	35.2	0.864
PBM V50Gy (%)	7.8	7.5	0.763
PBM V<10Gy (cc)	171	180	0.869
PBM V<20Gy (cc)	296	282	0.864
PBM V<30Gy (cc)	541	564	0.438
PBM V<40Gy (cc)	879	864	0.638
PBM V<50Gy (cc)	1268	1246	0.879

Abbreviations: BMI, body mass index; BSA, body surface area; G, grade; PBM, pelvic bone marrow; PTV, planning target volume; RTT, radiation treatment time; WBC, white blood cell
